# Amorphous, Smart, and Bioinspired Polyphosphate Nano/Microparticles: A Biomaterial for Regeneration and Repair of Osteo-Articular Impairments In-Situ

**DOI:** 10.3390/ijms19020427

**Published:** 2018-01-31

**Authors:** Werner E. G. Müller, Meik Neufurth, Shunfeng Wang, Maximilian Ackermann, Rafael Muñoz-Espí, Qingling Feng, Qiang Lu, Heinz C. Schröder, Xiaohong Wang

**Affiliations:** 1 ERC Advanced Investigator Grant Research Group at the Institute for Physiological Chemistry, University Medical Center of the Johannes Gutenberg University Mainz, Duesbergweg 6, 55128 Mainz, Germany; mneufurt@uni-mainz.de (M.N.); shunwang@uni-mainz.de (S.W.); qianglu1@uni-mainz.de (Q.L.); hschroed@uni-mainz.de (H.C.S.); 2 Institute of Functional and Clinical Anatomy, University Medical Center of the Johannes Gutenberg University Mainz, Johann Joachim Becher Weg 13, 55099 Mainz, Germany; maximilian.ackermann@uni-mainz.de; 3 Institute of Materials Science (ICMUV), Universitat de València, C/Catedràtic José Beltrán 2, Paterna, 46980 València, Spain; rafael.munoz@uv.es; 4 Key Laboratory of Advanced Materials of Ministry of Education of China, School of Materials Science and Engineering, Tsinghua University, Beijing 100084, China; biomater@mail.tsinghua.edu.cn

**Keywords:** long bone defects, bone marrow cells, inorganic polyphosphate, microparticles, bisphosphonates, *Runx2*, *Sox9*, cathepsin-K, tumor metastases, human mesenchymal stem cells

## Abstract

Using femur explants from mice as an in vitro model, we investigated the effect of the physiological polymer, inorganic polyphosphate (polyP), on differentiation of the cells of the bone marrow in their natural microenvironment into the osteogenic and chondrogenic lineages. In the form of amorphous Ca-polyP nano/microparticles, polyP retains its function to act as both an intra- and extracellular metabolic fuel and a stimulus eliciting morphogenetic signals. The method for synthesis of the nano/microparticles with the polyanionic polyP also allowed the fabrication of hybrid particles with the bisphosphonate zoledronic acid, a drug used in therapy of bone metastases in cancer patients. The results revealed that the amorphous Ca-polyP particles promote the growth/viability of mesenchymal stem cells, as well as the osteogenic and chondrogenic differentiation of the bone marrow cells in rat femur explants, as revealed by an upregulation of the expression of the transcription factors *SOX9* (differentiation towards osteoblasts) and *RUNX2* (chondrocyte differentiation). In parallel to this bone anabolic effect, incubation of the femur explants with these particles significantly reduced the expression of the gene encoding the osteoclast bone-catabolic enzyme, cathepsin-K, while the expression of the tartrate-resistant acid phosphatase remained unaffected. The gene expression data were supported by the finding of an increased mineralization of the cells in the femur explants in response to the Ca-polyP particles. Finally, we show that the hybrid particles of polyP complexed with zoledronic acid exhibit both the cytotoxic effect of the bisphosphonate and the morphogenetic and mineralization inducing activity of polyP. Our results suggest that the Ca-polyP nano/microparticles are not only a promising scaffold material for repairing long bone osteo-articular damages but can also be applied, as a hybrid with zoledronic acid, as a drug delivery system for treatment of bone metastases. The polyP particles are highlighted as genuine, smart, bioinspired nano/micro biomaterials.

## 1. Introduction

Cells can self-renew and/or produce differentiated cells basically through the following two ways: first, by proliferation of stem cells producing differentiated cells, by de- and subsequently by re-differentiation or by trans-differentiation, or second, by proliferation of pluripotent progenitor cells producing differentiated progeny cells or by different lineage-restricted progenitor cells each of them producing different differentiated cells [1]. Focusing on bone and cartilage, these tissues comprise only a small percentage of cells. While in bone, as hard calcified tissue, both inorganic and organic extracellular components form the structural scaffold, the semi-rigid cartilage is built, besides of water (75%), of organic macromolecules like aggrecan and proteoglycans (10%), and primarily of collagen fibers (20%). The cellular constituents of bone are osteoblasts (forming the bone material), osteocytes (present within the mature bone tissue and living as long as the organism itself), osteoclasts (degrading bone), and lining cells (involved in coupling bone resorption to bone formation), while the cells in cartilage comprise chondroblasts (perichondrial cells which develop to chondrocytes) and chondrocytes (producing and maintaining the cartilaginous matrix) [2]. Both cell lineages originate from osteochondro-progenitor cells, which arise from mesenchymal stem cells (MSC) in the bone marrow and differentiate into osteoblasts or chondrocytes, depending on the signaling molecules they are exposed to [3]. Due to the inherent potential of MSCs to differentiate either into bone, cartilage, or fat tissue, recent studies have shown their healing capability by improving angiogenesis and preventing fibrosis [4]. In addition, they exhibit a positive role in tissue repair and tissue regeneration, especially in the field of bone-healing problems and early stages of local bone defects, as well as osteo-articular diseases [5]. These bone/osteo-articular impairments are characterized by a slowly decreasing bone metabolism, as well as cartilage metabolism that is generalizing and progressing. It is the physiological polymer polyphosphate (polyP) that induces the repair processes of both bone and cartilage in an anabolic way.

Recently, this inorganic polymer, polyP, has been identified to deliver metabolic energy and to elicit morphogenetic signals in mammalian cells [6,7]. Those signals promote and accelerate cell growth and differentiation and upregulate the steady-state-expression of genes involved in differential gene expression [8]. polyP is found as short-chain (2–10 mer) and medium- (until 100 units) to long-chain molecules [9]; physiologically, polyP molecules with a chain length between 40 and 60 are found. In animal systems and also in humans, polyP is abundantly present both intra- and extracellularly [10,11]. The amount of polyP in mammalian cells depends on the physiological state of the cells. As summarized [12,13], in non-stimulated osteoblasts the concentration of polyP is around 500 μM (estimated as orthophosphate residues), while in human plasma cells, it is 10-fold lower. In the blood platelets the level of polyP is comparably high (in the millimolar range). Generally, in mammalian organelles, polyP has been determined within the μM range. In this context, it is interesting to mention that the autologous blood platelets gel has been described to accelerate the healing process of osteochondral, muscle, tendon, and ligament lesions [14].

The polyP polymer can be applied to cells in two forms, either as soluble sodium polyP (“Na-polyP”) or as particulate Ca-polyP nano/microparticles (“Ca-polyP-MP”). Both polyP preparations have been described to act anabolically on osteoblasts, or osteoblast-like cells in vitro [15,16]. The soluble polyP is likely to interact with the cell surface G-protein-coupled receptor P2Y1 and to initiate an intracellular signal transduction pathway [17]. In contrast, the Ca-polyP-MP are taken up by clathrin-dependent endocytosis [10] and do not affect the Ca^2+^ level. It is important to mention that both the soluble and the particulate polyP enhance the extracellular and/or intracellular ATP pools [18]. Concerning the intracellular ATP level, the activation of purinergic receptors is known to cause an increase in the intracellular ATP content [19]. Recently, it has been disclosed that polyP, after enzymatic hydrolysis via the alkaline phosphatase (ALP) and concurrent release of metabolic energy, causes an increased phosphorylation of adenylate nucleotides most likely via the adenylate kinase [20]. Hence, polyP acts as an energy store for the formation of both extra- and intracellular ATP by transferring the Gibbs free energy, released during the ALP-mediated hydrolysis of the high-energy phosphoanhydride bonds of polyP. In contrast to the effect on osteoblasts, polyP inhibits the progression of osteoclast-like RAW 264.7 cells to functional osteoclasts. We could demonstrate that the number of TRAP^+^ (tartrate-resistant acid phosphatase positive) cells is significantly reduced by polyP after induction with RANKL (receptor activator of the NF-κB ligand) [21].

In metazoan cells, and also in human cells, polyP is encapsulated in acidocalcisomes in an amorphous state [22]. Taking nature as a model, we succeeded to fabricate in a biomimetic way amorphous nano/microparticles and nanoparticles of Ca-polyP that retain the morphogenetic function of the soluble polymer. This process became possible by co-precipitating Na-polyP with CaCl_2_ in a 1:2 weight ratio at alkaline condition. At those super-stoichiometric conditions, a crystalline deposition could be prevented. This process allows also the entrapping of anionic drugs, like the bisphosphonate zoledronic acid (ZOL), in the polyP particles via Ca^2+^ ionic linkages, as shown here. ZOL had been described to be a tumor-specific drug, acting especially on bone tumor metastases. This (intravenously applied) bisphosphonate is used for the treatment of cancer bone metastases, besides surgical resection [23,24,25].

Considering the beneficial effects of polyP, fabricated as micro/nanoparticles, on bone anabolic pathways in vitro and in vivo [8,10,11], we tried to get a first hint if those particles affect also MSC, present in bone marrow of rat femurs, towards an osteogenic or chondrogenic effect. In our studies, we removed the femurs from mice and opened the cortical bone protective shell around the spongy bone tissue. To distinguish between the two cell lineages originating from MSC, the osteogenic and chondrogenic one, we did not select a late molecular marker, e.g., the expression ratio between *collagen 2A1* and *collagen 1A1* [26], but the two transcription factors, *Runx2*, as an indicator gene for the expression towards the osteogenic lineage [27], and *Sox9*, which is required for chondrocyte differentiation [28,29].

In parallel to the development of the macrophage lineage, the osteoclast cells maturate. Focusing on the latter cell type, the tartrate-resistant acid phosphatase (TRAP) has been originally considered as a marker protein and gene for osteoclasts [30,31]; this enzyme is now approved as a widespread molecule, which is critically expressed during the development and growth in distinct zone of the long bone [32] and even involved in osteoblast gene expression [33]. However, cathepsin-K is an essential enzyme, required for normal bone resorption [34]. In vivo bone resorption depends upon the synthesis of cathepsin-K in osteoclasts. The manifestations of bone metastases are either osteolytic, bone destructive, or osteoblastic, bone forming-sclerotic [35]. Bisphosphonates like ZOL are hydrolysis-resistant structural analogs of pyrophosphate; they tightly bind to bone and are strongly internalized by osteoclasts, turning them to cells that are lacking the property of bone resorption.

As long bone samples, the femurs from mice have been selected as a model [36]. The specimens could be obtained in a straightforward way and allowed also to collect the bone marrow in a simple and gentle manner. They were found to contain metabolically active, bone forming osteoblasts and bone resorbing osteoclasts together with the stem cell niches, required for the maintenance of hematopoietic and lymphoid cell populations [37]. As outlined under “Results”, polyP particles in the femur model anabolically affect key regulatory molecules directing the MSC into both bone (*SOX9*, differentiation towards osteoblasts) and cartilage formation (*RUNX2*, chondrocyte differentiation). In turn, this polymer, if fabricated in the form of amorphous particles, appears to be applicable for the repair of osteo-articular impairments and restorations.

## 2. Results

### 2.1. Nano/Microparticles with polyP and ZOL

**Figure 1:**
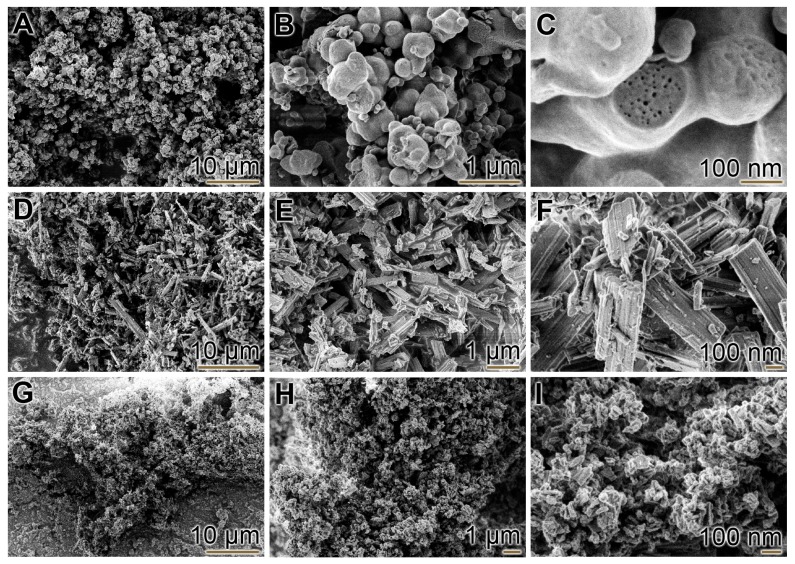
Morphology of the polyP and ZOL particles; SEM. Images from (**A**–**C**) amorphous “Ca-polyP-MP”, (**D**–**F**) crystalline “Ca-ZOL-MP”, and (**G**–**I**) amorphous hybrid “Ca-polyP-ZOL-MP”. ZOL, zoledronic acid.

Particles from both polyP and ZOL were prepared by incubation with CaCl_2_. In a previous study [16], we could establish that polyP, if added to CaCl_2_ in a 1:2 weight ratio (polyP:CaCl_2_), precipitated as amorphous nano/microparticles. This procedure was also followed here. Particles (“Ca-polyP-MP”) with an average diameter of 280 ± 120 nm (*n* = 50) were formed under the conditions described under “Materials and Methods” (Figure 1A–C). Those particles are not completely solid, but traversed by ≈10 nm large canals (Figure 1C). In contrast to those globular particles, the particles prepared from ZOL (“Ca-ZOL-MP”) are monoclinic crystals that usually have a square-shaped to octagonal centrosymmetric morphology. The length of the minor axis is ≈500 nm, and those of the major axes are of ≈2–3 µm (Figure 1D–F). Those crystals have been described before as crystalline, rod-like particles with ≈80 × ≈80 × ≈1000 nm in dimensions [38].

Hybrid composite polyP/ZOL hybrid particles (“Ca-polyP-ZOL-MP”), with a mesocrystal-like morphology, were prepared by dissolving Na-polyP and ZOL in an equi-weight ratio at pH 10 followed by a subsequent precipitation step with a surplus on CaCl_2_. The resulting particles with an average diameter of 60 nm (Figure 1G–I) were collected, and the morphology was found to be rhombic-like but with axes that are very close to all three directions. The dug loading was computed by determining the percentage of ZOL in the particles versus the concentration in the surrounding medium. It was found to be 31.2 wt % of ZOL in the “Ca-polyP-ZOL-MP”.

### 2.2. Composition and Phases of the Particles

**Figure 2:**
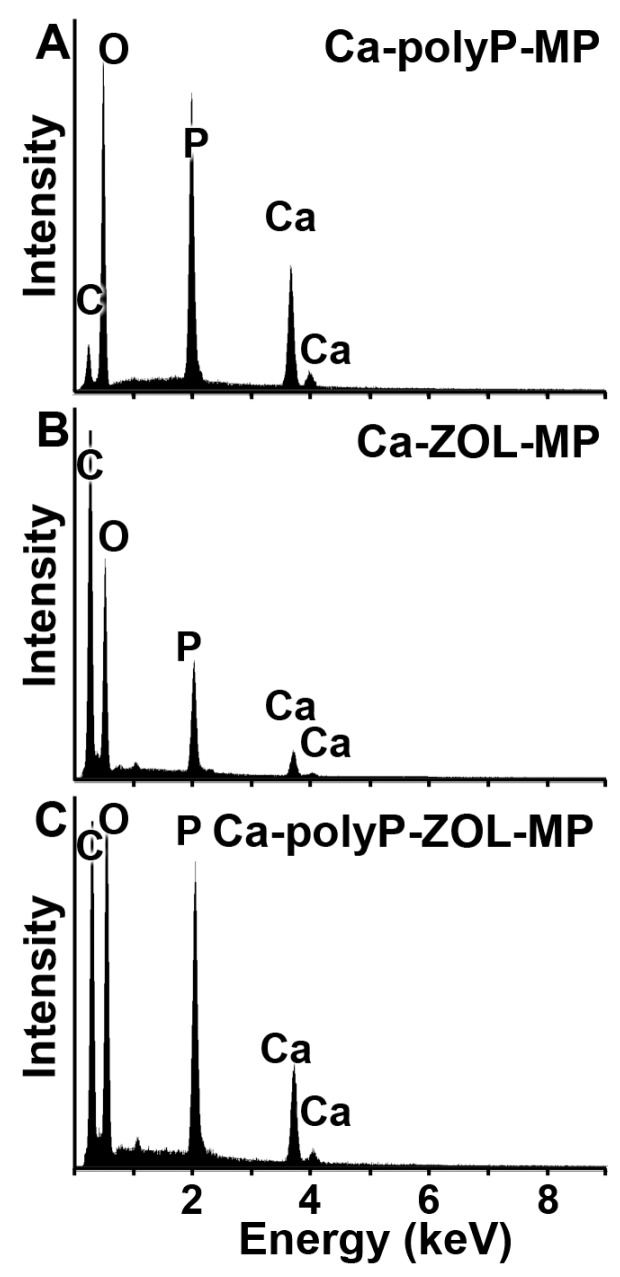
EDX spectra for (**A**) “Ca-polyP-MP”, (**B**) “Ca-ZOL-MP”, and (**C**) “Ca-polyP-ZOL-MP”. The respective signals for the different atoms are marked. EDX, energy dispersive X-ray.

The particles were analyzed by energy dispersive X-ray (EDX) spectroscopy. As expected [16], the EDX spectrum for “Ca-polyP-MP” shows major signals for O, P, and Ca and a very minor peak that appears with C (Figure 2A). In contrast, in the “Ca-ZOL-MP” particles, in addition, a strong signal for C is present that reflects the carbon backbone of ZOL (Figure 2B). A superposition of the two spectra, “Ca-polyP-MP” and “Ca-ZOL-MP”, is recorded for “Ca-polyP-ZOL-MP” with an almost equal peak for C and P (Figure 2C).

**Figure 3:**
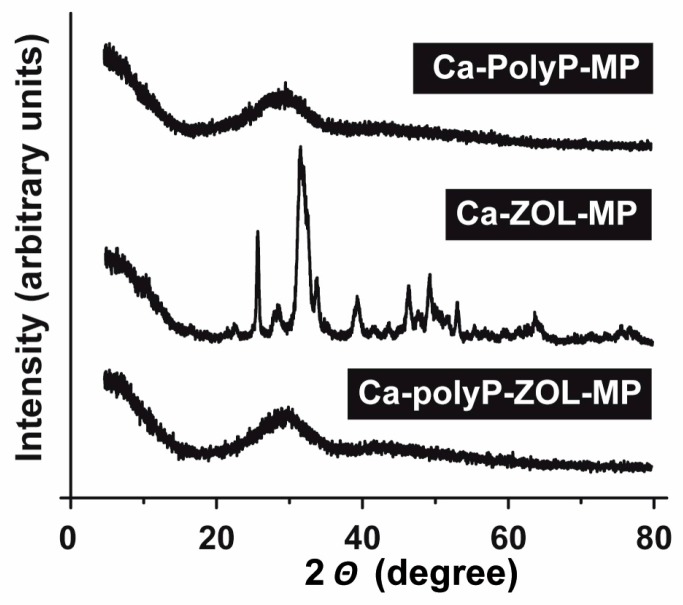
XRD pattern obtained from (**above**) “Ca-polyP-MP”, (**middle**) “Ca-ZOL-MP”, and (**below**) “Ca-polyP-ZOL-MP”. The characteristic signals in the diffractogram from “Ca-ZOL-MP”, which match those of ZOL, are striking. XRD, X-ray diffraction.

X-ray diffraction (XRD) patterns for “Ca-polyP-MP” and “Ca-polyP-ZOL-MP” show a clear amorphous phase, with a broad amorphous halo from 20° to 40° (Figure 3). In contrast, the spectrum for the “Ca-ZOL-MP” reflects a crystalline phase with peaks at 2θ values of 27.7°, 33.5°. This diffractogram shows characteristic signals, also recorded previously for ZOL crystals [39].

### 2.3. Dose-Finding Assay with Human Mesenchymal Stem Cells

**Figure 4:**
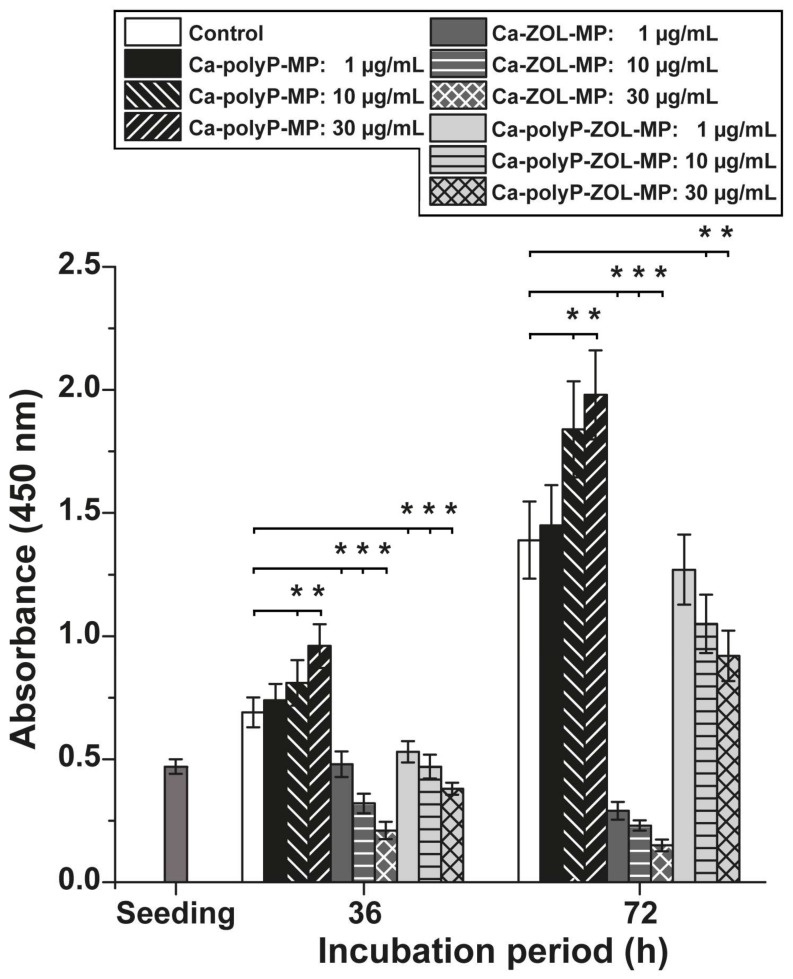
Effect of the different concentrations of particles, either “Ca-polyP-MP”, “Ca-ZOL-MP”, or “Ca-polyP-ZOL-MP” the viability/growth of MSC. The number of viable cells was determined by the XTT assay (A_450_ values). The incubation period was 36 h and 72 h, respectively. Ten parallel assays were performed, and the mean values (±SD) were determined. The significance correlations are determined with respect to the controls (no particles added); they are marked with rectangular brackets; (* *p* < 0.001) between the indicated two values. The concentrations of the particles for the respective test samples are given in the box.

For the determination of the biological activities of polyP and ZOL on cells in the femur explants system, we performed dose range finding pilot toxicology studies with human mesenchymal stem cells (MSC) in vitro. The cells were incubated for 36 h and 72 h, respectively; then the XTT assay for cell viability and growth was performed. The data show that “Ca-polyP-MP” at concentrations of 10 µg/mL and 30 µg/mL already after 36 h significantly increase the viability of the cells, compared to the controls without any additional compound (Figure 4). These statistical significance values even increase after the total (72 h) incubation period; the A_450_ absorbance value increases to 142% (at 30 µg/mL). Inversely proportional is the effect of “Ca-ZOL-MP”; these particles strongly inhibit the viability already after 36 h at all concentrations tested. Interesting is the finding that the hybrid particles “Ca-polyP-ZOL-MP” display a moderate inhibitory activity that reaches ≈55% of the viability, seen in the controls (at 30 µg/mL and an incubation period of 36 h; Figure 4). We suspect that the reduction of the cytostatic activity can be attributed to the encapsulation of the drug into polyP particles, which allow only a slow release. We conclude that a concentration of 30 µg/mL of the respective particles is suitable for the gene expression studies.

### 2.4. Incubation of Rat Femur Explants

**Figure 5:**
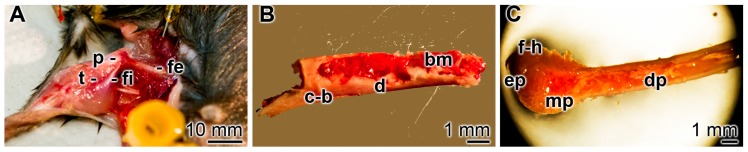
Dissection and collection of mice femurs. (**A**) Immediately after sacrificing, the animals and the femurs were dissected and collected. In addition to the exposed femur (fe), the tibia (t), patella (p), and fibula (fi) are visible; (**B**) the explant, measuring up to 25 mm in length and 2 mm in diameter, shows the compact bone zone (c-b) and the diaphysis (d), and exposes the bone marrow (bm); (**C**) after incubation in DMEM medium/FBS for up to 7 days, the specimens were collected and processed for bone marrow cell isolation. On the pictured specimen the femoral head (f-h), the epiphysis (ep), the metaphysis (mp), and the diaphysis (dp) can be distinguished.

Mice femurs were prepared immediately after sacrificing the animals. As outlined under “Materials and Methods”, the animals were rapidly dissected after sacrifice and the femurs of the hind legs were exposed and collected (Figure 5A). They were immediately submersed in DMEM medium/FBS (Figure 5B). After an incubation period of, usually, 3 to 7 days, the bone samples were used for the analyses (Figure 5C). From these samples, the bone marrow could be isolated preserving the sterility of the bone marrow space. Those bone marrow samples were found to be rich in osteoclasts and osteoblasts [36].

### 2.5. Gene Expression Studies: Effect on Osteogenic and Chondrogenic Differentiation in Bone Marrow Cells

In a first series of experiments, the effects of the polyP and ZOL particles on the osteogenic and chondrogenic differentiation of the cell lineages in the bone marrow cells were studied. As marker genes, the steady-state-expressions of *Sox9* (marker for the chondrocyte differentiation) and of *Runx2* (marker for osteogenic lineage) were determined.

**Figure 6:**
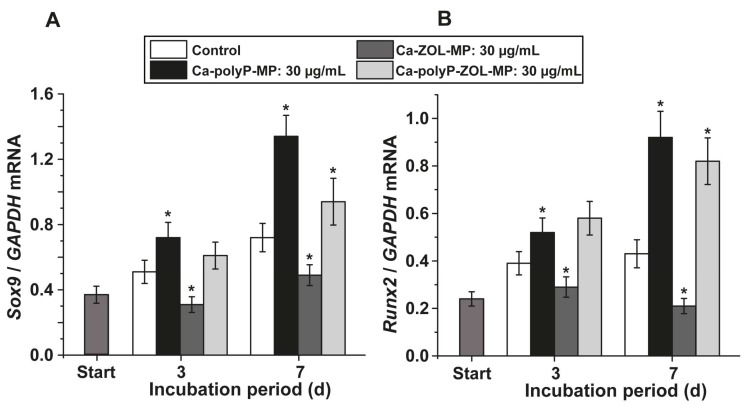
Effect of the compounds on the expression of osteogenic and chondrogenic differentiation markers in bone marrow cells. The cultures were supplemented with 30 µg/mL of “Ca-polyP-MP”, “Ca-ZOL-MP”, or “Ca-polyP-ZOL-MP” on bone marrow cells in mouse femurs; in the controls the explants received no additional compound. The cells were incubated for 3 days or 7 days, then the RNA was isolated from the samples and the steady-state-expression of the transcripts of (**A**) *Sox9* and (**B**) *Runx2* was quantified by qRT-PCR and correlated to the expression of the *GAPDH* house-keeping gene. Standard errors of the means are shown (*n* = 6 experiments per time point). The significant differences between the values in the controls and the respective treated samples are indicated with asterisks; * *p* < 0.01.

*Sox9* expression: At the beginning of the incubation, after 3 days, the expression level of *Sox9* measures 0.37 expression units (correlated to the reference *GAPDH* expression level); this level increases both in the controls, but especially in the experiments with “Ca-polyP-MP” significantly later during the incubation period, 7 days (Figure 6A). In contrast, ZOL particles, “Ca-ZOL-MP”, strongly and significantly inhibit the accumulation of transcripts in bone marrow cells already after a 3 days incubation. This low level drops further down to 37%, with respect to the polyP exposed cultures, after the longer (7 days) incubation period. If this chemotherapeutic drug is encapsulated into polyP nano/microparticles, the inhibitor effect of ZOL is strongly reduced; the level drops to 70% if correlated to the expression under polyP microparticles.

*Runx2* expression: A similar, even more distinct response level is seen if, in the same cells, the expression of *Runx2*, an osteogenic differentiation marker, is assayed (Figure 6B). Again with “Ca-polyP-MP”, the expression level strongly increases during the 3 days and 7 days incubation period; after 7 days, the expression level increases with respect to the expression reference gene *GAPDH* and is correlated to the controls to 2.1-fold. The expression of *Runx2* after exposure to “Ca-ZOL-MP” drops significantly after 3 days, and even more after 7 days. Testing studies with “Ca-polyP-ZOL-MP” disclosed that this hybrid material causes almost the same expression level as “Ca-polyP-MP”, which lacks the drug.

### 2.6. Gene Expression Studies: Effect on Osteoclastic Differentiation

The expression level of the *cathepsin-K* gene is considered as a reliable marker for the function of osteoclasts, while the *TRAP* gene expression level reflects an overall proliferation capacity of osteoclasts and osteoblasts.

**Figure 7:**
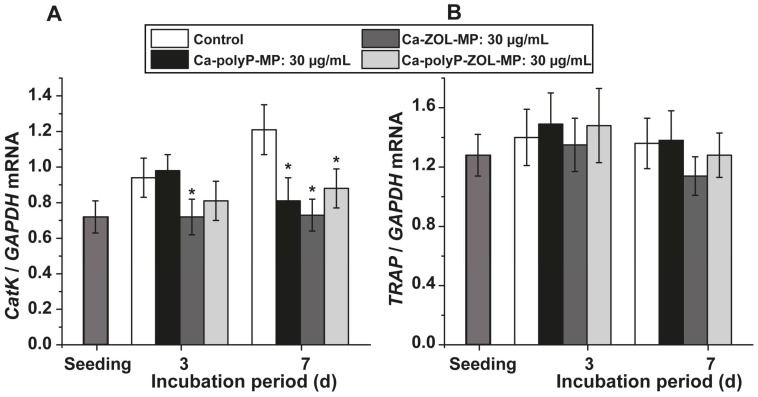
Alteration of the expression in bone marrow cells using (**A**) the established osteoclastic differentiation marker *cathepsin-K* and (**B**) the level of *TRAP*, which reflects both the osteogenic and the osteo-clastogenic proliferation state of the cells; * *p* < 0.001.

*Cathepsin-K* expression: After exposure of the femur explants to “Ca-polyP-MP”, “Ca-ZOL-MP”, or “Ca-polyP-ZOL-MP”, no significant (or only a small) change in the steady-state-expression is seen both for *cathepsin-K* expression and *TRAP* expression in bone marrow cells after a 3 days incubation period, if correlated to the level in the controls (Figure 7A,B). However, after the longer 7 days incubation time, the expression of *cathepsin-K* drops significantly in the presence of “Ca-ZOL-MP” and also in the presence of “Ca-polyP-MP” and “Ca-polyP-ZOL-MP” (Figure 7A).

### 2.7. Mineralization by Bone Marrow Cells in the Presence of polyP and ZOL

**Figure 8:**
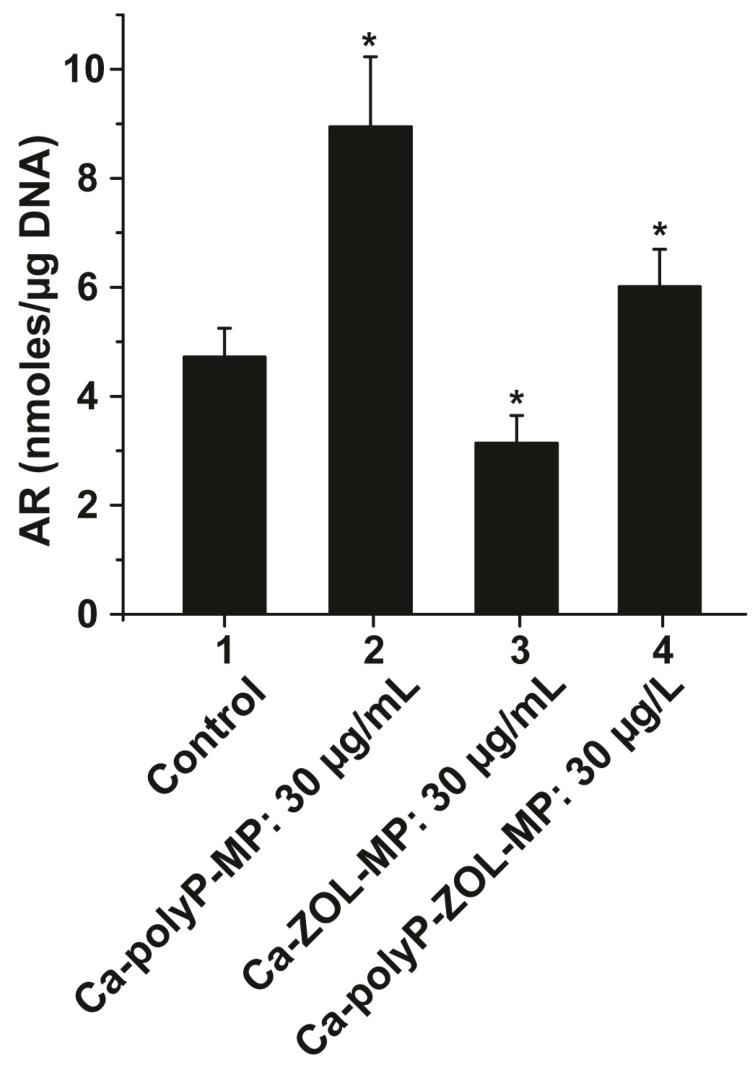
Influence of the particles (30 µg/mL of “Ca-polyP-MP”, “Ca-ZOL-MP”, or “Ca-polyP-ZOL-MP”) on bone marrow cells present in femurs on the extent of biomineralization during a 5 days incubation in DMEM medium/FBS. After termination of the experiments, the bone marrow cells were carefully removed from the femurs and quantitatively assayed for mineralization with Alizarin Red S. The reintroduced red color is measured at 405 nm applying the spectrophotometric assay. The amount of bound Alizarin Red S is given in nmol. Values were normalized to 1 µg of DNA in the samples; The significant differences are indicated with asterisks; * *p* < 0.01.

Mouse bone marrow cells were cultivated in femur explants for 5 days in the absence (control) or presence 30 µg/mL of “Ca-polyP-MP”, “Ca-ZOL-MP”, or “Ca-polyP-ZOL-MP”. The mineralization was determined quantitatively with the Alizarin Red S dye (Figure 8). In the absence of any additional component, the extent of biomineralization was low, with 4.72 ± 0.53 nmol bound Alizarin Red S·µg DNA^−1^. After exposure of the femurs to 30 µg/mL of “Ca-polyP-MP” or “Ca-polyP-ZOL-MP”, the level increases to 8.95 ± 1.28 or to 6.01 ± 0.69 nmol·µg DNA^−1^. However, after exposure to 30 µg/mL of “Ca-ZOL-MP”, the Alizarin Red S-positive material drops significantly to 3.14 ± 0.51 nmol·µg DNA^−1^.

## 3. Discussion

The repair of long bone defects is a complex challenge, since this task involves different hard tissues as well as non-calcified tissue, e.g., cartilage. The common denominator of those tissues is their bulky extracellular matrix (ECM), which is comparably poor in cells. In this ECM complex, organic macromolecules (collagens, non-collagenous glycoproteins, hyaluronan, and proteoglycans) and inorganic deposits (hydroxyapatite) are present that are organized by both self-assembling processes and mechanical, energy-dependent, forces [40]. This fine-tuned dynamic processes depend on a dynamic equilibrium of supply and demand of nutrients, growth factors, and signaling molecules in order to keep running the complex mechanisms that control turnover and remodeling of the ECM and the bone.

The physiological polymer, polyP, is most likely the common denominator for extracellular metabolic energy and releases Gibbs free energy (ΔG) during hydrolysis of the acid anhydride linkages within the polymer chain under formation of ADP and ATP from AMP and/or ADP [10,20]. With this discovery, a new avenue has been opened towards an understanding of the energetics during bone and cartilage formation, resorption, and remodeling [41]. polyP is abundantly present in blood platelets, serum, and the synovial fluid [42]. With the discovery of the technology to prepare particles or spheres, consisting of Ca-polyP nano- or microparticles, in a biomimetic process [16], which even allows functionalization, e.g., with hyaluronic acid [43], innovative development lines could be opened that are (likely) suitable for the introduction as orthopedic implants. The polyP material turned out to be applicable both for soft, semi-hard, and hard tissue, from cartilage [43] to bone repair in vitro and in vivo [44,45]. The unique feature of polyP is that this physiological polymer can be hardened to meet either the characteristics of cartilage or bone [43], just by varying the percentage of polyP in the implant or the concentration and duration of exposure to Ca^2+^ [10]. In turn, it was not surprising that polyP could also be used as a functional ingredient in bio-inks useful for bio-printing of cells to tissue-like units [46].

In the present study, two problems have been addressed: firstly, finding out if polyP is in any kind or formulation, ranging from injection solution to implant material, either to the bone marrow or to the bone, which is a promising route, especially for the repair of long bone damages; and secondly, developing a strategy for the encapsulation of a cytostatic/cytotoxic agent into the polyP particles. For this work, femur explants were prepared from mice. The cells in those bone/bone marrow fragments retain, for a short period of incubation, largely physiological conditions. The polyP polymer was applied as a Ca^2+^-polyP salt that remained amorphous, the characteristics that we consider to be essential for larger scale regeneratively active implants. This notion is supported by the finding that crystalline hard tissues have also to pass the amorphous phase [47]. Those amorphous polyP particles were found to support growth/viability of bone marrow cells at concentrations around 30 µg/mL. By application of the qRT-PCR technique, the experimental data show that “Ca-polyP-MP” upregulate the expression of the two master transcription factors, *SOX9* and *RUNX2*, indicative for differentiation of mesenchymal, stem cell-derived osteochondroprogenitors. These transcription factors are essential for chondrogenesis and osteogenesis [48]. In retrospect, this result is supported by in vitro and in vivo findings showing that platelet-rich plasma (PRP) aids repair of tissue and its regeneration [49]. This property of PRP can be partially attributed to the blood-derived growth factors, associated with the blood platelets. However, since polyP is a major component, found in blood platelets, it is reasonable to accept that this inorganic polymer plays an important role in the activity of the platelets to induce regeneration [7]. When adding PRP, in addition to medium/serum, to the cells, an increased expression *Runx2* [49] and *Sox9* has been observed [50]. PRP, if combined with β-tricalcium phosphate scaffolds, attracts in vivo osteoblasts [51].

The anabolic pathways during bone formation are controlled, balanced, and dynamically counter-balanced by bone resorption [52]. Blood platelets elicit anabolic but not catabolic signals during bone and cartilage repair [53], including articular cartilage regeneration [54]. Applying the molecular marker genes *cathepsin-K* and *TRAP,* it has been documented that PRP down-regulates the steady-state-expression of both genes [55]. In the present study, polyP, added as particles to femur explants, significantly reduced the expression of the *cathepsin-K* gene after 7 days, while leaving the expression of *TRAP* unaffected. It could well be that the effect of polyP on *TRAP* expression is different depending on the region of the osteocytes/osteoblasts in the bone marrow; a regional different quantitative and qualitative composition of the cells in the bone marrow of different bones has been described [56].

In a second task, we applied polyP as a biodegradable carrier for the fabrication of nano/microparticles with embedded chemotherapeutic agents. Among all tissues and besides lung and liver, bone is one preferred site of metastatic disease. As an example, ≈70% of patients who die with breast or prostate cancer develop bone metastases. A similar high prevalence exists for thyroid, lung, and kidney cancers [57,58]. Most bone metastases originate from the diaphysis and the metaphysis of long bones. At present, those larger defects are treated, besides with chemotherapy, by transplantation with all their limitations, with respect to material availability and risk of infection. After surgical removal of the tumor in the bone region, the remaining bone cavity can be filled with polyP particles processed via Ca^2+^ ionic linkages with the standard bone tumor drug zoledronic acid. Those support enclosures will not only produce mechanical stability but also bind the drug, which undergoes slow release.

We propose, in the present study, a new, promising avenue for fabricating an implant/scaffold material suitable for the substitution of bone lesion which, simultaneously, is filled to a high yield with a chemotherapeutic agent, here zoledronic acid. The scaffold constituent studied, polyP, is morphogenetically active, meaning that it elicits significant anabolic stimulus for bone growth, like expression of the *ALP* gene [15], and as shown in the present study, promotes the differentiation of bone marrow cells/MSC towards the osteogenic or chondrogenic direction through activation of *Sox9* and *Runx2* transcription factors. Interesting enough is also the anti-catabolic activity of polyP, which was found to reduce the level of cathepsin-K, an enzyme involved in bone resorption. With regard to those properties, polyP shares important characteristics with blood platelets, i.e., it acts on bone and cartilage defects. We succeeded in introducing a technology to fabricate amorphous particles that retain those bone pro-anabolic properties [10] and undergo enzymatic hydrolysis via the ALP under release of metabolic energy and orthophosphate; the latter metabolite becomes channeled in the bone during its development [59]. The polyanionic polyP, added together with the likewise anionic bisphosphonate ZOL, forms hybrid nano/microparticles that release both the morphogenic stimuli elicited from polyP and the cytostatic/cytotoxic effects originating from ZOL. It is worth mentioning that those hybrid particles promote biomineralization in bone marrow cells/MSC present in their physiological microenvironment (femur explants), as proven here by application of the Alizarin red-based assay. Considering the fact that ZOL is a well-proven chemotherapeutic agent used to treat cancers that have spread to the bones [60], polyP particles enriched with ZOL appear to be a promising multi-functional hybrid scaffold material to be used as a delivery system for drugs directed against bone metastases, as well as for treatment of osteo-articular disorders.

## 4. Materials and Methods

### 4.1. Materials

Na-polyphosphate (Na-polyP) with an average chain length of 40 phosphate units was from Chemische Fabrik Budenheim (Budenheim, Germany). Zoledronic acid monohydrate was obtained from TCI Germany (#Z0031; Eschborn, Germany).

### 4.2. Preparation of Nano/Microparticles

Calcium polyP (“Ca-polyP-MP”) microparticles were prepared from Na-polyP as described [16]. In brief, 2.8 g of CaCl_2_·2H_2_O (#223506; Sigma-Aldrich, Taufkirchen, Germany) were dissolved in 25 mL of distilled water and added dropwise to 1 g of Na-polyP in 25 mL distilled water at room temperature. Importantly, the suspension was kept at pH 10; then, it was stirred for 12 h. The nano/microparticles formed were collected by filtration, washed with ethanol, and dried at 50 °C.

Calcium zoledronic acid nano/microparticles (“Ca-ZOL-MP”) were fabricated in an analogous procedure. The preparation started with 1 g of zoledronic acid (ZOL), dissolved in 50 mL of distilled water. The pH of the suspension was adjusted to pH 7 (using NaOH). Then, 2.8 g of CaCl_2_·2H_2_O, dissolved in 50 mL water, was added dropwise. Again, the pH was adjusted to 7 and the suspension was stirred overnight. Finally, the particles were washed three times with water, followed by a washing step in ethanol. Then, the particles were dried in an oven for overnight at 60 °C.

Calcium polyP-zoledronic acid hybrid nano/microparticles (“Ca-polyP-ZOL-MP”), containing a mixture of polyP and ZOL, were prepared as follows. Na-polyP, at a concentration of 0.5 g in 100 mL distilled water (pH 10), was added to 0.5 g of ZOL, dissolved in 100 mL water (pH 7). Then, 2.8 g of CaCl_2_·2H_2_O, dissolved in 50 mL water, was added dropwise. Crucial is that during this process, the pH remained at 10 by addition of NaOH. After stirring overnight, the particles were filtrated, washed in water, and then washed in ethanol. Then, the particles were dried at 60 °C.

The loading efficiencies of the nano/microparticles composed of ZOL were determined as described [38]. Accordingly, a standard curve was established by measuring the absorbance of five defined concentrations of ZOL in 0.1 M HCl. The concentration of drug in the solution was determined at 215 nm. The dug loading was computed by determining the percentage of ZOL in the particles versus the concentration in the surrounding medium.

### 4.3. Electron Microscopy, Energy Dispersive X-ray, and X-ray Diffraction Spectroscopy

Scanning electron microscopic (SEM): Imaging was performed using a Hitachi SU-8000 electron microscope (Hitachi High-Technologies Europe, Krefeld, Germany).

EDX analysis: The experiments were performed with an EDAX Genesis EDX System attached to a scanning electron microscope (Nova 600 Nanolab, FEI, Eindhoven, The Netherlands) operating at 10 kV with a collection time of 30–45 s.

XRD analysis: Diffractograms of dried powder samples were registered with a D8 Avance A25 diffractometer (Bruker, Billerica, MA, USA) with monochromatic Cu K-α radiation [61].

### 4.4. Human Mesenchymal Stem Cells

Human mesenchymal stem cells (MSC), isolated from normal (non-diabetic) adult human bone marrow of healthy volunteers, were purchased from Lonza (Cologne, Germany) and cultivated [62]. The cells were maintained in 75 cm^2^ flasks and grown in α-MEM (#F0915; Biochrom, Berlin, Germany), supplemented with 20% FCS (Life Technologies, Darmstadt, Germany) together with 0.5 mg/mL of gentamycin, 100 units/mL of penicillin, 100 μg/mL of streptomycin, and 1 mM pyruvate. The cultures were incubated in a humidified incubator at 37 °C.

For cell viability, the MSC cultures were inoculated with 1 × 10^4^ cells per well (48 well plates; #CLS3548; Sigma-Corning, Taufkirchen, Germany) in a total volume of 0.5 mL. The cultures remained untreated (controls), or were treated with different concentrations (1 µg/mL to 30 µg/mL) of “Ca-polyP-MP”, “Ca-ZOL-MP” or “Ca-polyP-ZOL-MP”. Then, the XTT assay was performed.

### 4.5. Cell Proliferation/Cell Viability Assay

The XTT Cell Proliferation Kit II [2,3-bis-(2-methoxy-4-nitro-5-sulfophenyl)-2H-tetrazolium-5-carboxanilide] from Roche (Mannheim, Germany) was used as outlined [63]. The cell growth/metabolic activity was determined by the colorimetric method, based on oxidation of the tetrazolium salt. The absorbance was determined at 450 nm and subtracted by the background values (500 nm). Routinely, the viable cells were determined after 36 h and 72 h, respectively.

### 4.6. Preparation and Culture of Femur Explants

The femur specimens were obtained from cadaver of male C57BL/6 mice (Charles River Laboratories, Sulzfeld, Germany). They were provided by the Institute of Functional and Clinical Anatomy, University Medical Center of the Johannes Gutenberg University, Mainz, Germany, following the ethical guidelines of the University Medical Center Mainz. The permission was obtained from the Ethical Committee of the “University Medical Center of the Johannes Gutenberg University Mainz”—No. 12-05-2015. The bones from the hind-legs were taken from the animal, following a published procedure [36,64]. After opening of the experimental animals, the femur with the patella was taken and overextended over the knee joint. By gentle rotation of the scissor, the condyles, the patella, and the epiphysis were removed and the metaphysis was isolated. The associated muscle and connective tissue were removed as far as possible and the femur was collected. Prior to incubation, the bone was longitudinally sliced.

The processed samples were incubated in DMEM medium (plus pyruvate, l-glutamine (#11995-065; Life Technologies, Darmstadt, Germany)), supplemented with 10% fetal bovine serum (heat inactivated; FBS; Life Technologies) [65]. The bone marrow was isolated by low speed centrifugation [36]. The femur explants were incubated, usually for 3 days or 7 days, in the absence of any additional compound (control) or in the presence of “Ca-polyP-MP”, “Ca-ZOL-MP”, or “Ca-polyP-ZOL-MP”, at a concentration of 30 µg/mL. Then, the bone marrow cells were isolated and used for the PCR analyses.

### 4.7. Quantitative Real-Time Polymerase Chain Reaction

The quantitative real-time polymerase chain reaction (qRT-PCR) was performed as described [66]. The femur explants from mice were incubated as described above for the indicated period of time. Then, the bone specimens were removed from the medium/serum and the bone marrow was isolated by centrifugation. This material was used for the expression studies using the following primers pairs: for the expression in the chondrocyte differentiation lineage, the transcription factor *Sox9* (*Mus musculus* SRY (sex determining region Y)-box 9 [67]), Fwd: 5′-GTACCCGCATCTGCACAAC-3′, and Rev: 5′-TCCACGAAGGGTCTCTTCTC-3′; and, for the expression in the osteogenic lineage one, *Runx2* (*Mus musculus* runt related transcription factor 2 [68]), Fwd: 5′-CCGCACGACAACCGCACCAT-3′, and Rev: 5′-AGCCACCAAGGCTGGAGTCTT-3′. As a reference, the expression of *GAPDH* (NM 001289726.1; glyceraldehyde 3-phosphate dehydrogenase [69]) was determined with the following primer pair; Fwd: 5′-GGTGAAGGTCGGTGTGAACG-3′ and Rev: 5′-CTCGCTCCTGGAAGATGGTG-3′. The RNA was extracted from the cells, which were then used for qRT-PCR in the iCycler (Bio-Rad, Hercules, CA, USA). The mean *C_t_* values and efficiencies were calculated by the iCycler software (Bio-Rad); the estimated PCR efficiencies were in the range of 93%–103%.

To check the effect of the compounds on the osteoclasts and their precursor cells in mice femur explants, we determined the expression of the *cathepsin-K* [70]. The following primer pair, which matched with the cDNA, was applied (CatK; accession number: BC046320); Fwd: 5′-TTAATTTGGGAGAAAAACCT-3′ and the Rev: 5′-AGCCGCCTCCACAGCCATAAT-3′. The overall expression of the gene expressing the tartrate-resistant acid phosphatase (TRAP; AI323530) was followed with the following primer pairs; Fwd 5′-GCTTTTTGAGCCAGGACAGC-3′ and Rev: 5′-CAGCCCAAAATGCCTCGA-3′ [33].

### 4.8. Mineralization by Cells in Femur Explants

For the mineralization studies, femur explants were incubated for 5 days in DMEM medium/FBS. Then, they were collected and the bone marrow was removed from the bone by centrifugation. Then, the minerals were extracted with 10% acetic acid and neutralized (ammonium hydroxide); Alizarin Red S was added. The reintroduced red color is measured at 405 nm by applying the spectrophotometric assay [71]. The amount of bound Alizarin Red S is given in nmol. Values were normalized to 1 µg DNA in the samples.

### 4.9. Further Analytical Determination

DNA contents in the bone marrow sample were determined using the PicoGreen method [72] with calf thymus DNA as a standard.

### 4.10. Statistical Analysis

After verification that the respective values follow a standard normal Gaussian distribution and that the variances of the respective groups are equal, the results were statistically evaluated using the independent two-sample Student’s *t*-test [73].

## 5. Conclusions

In the present study, we used femur explants as a model to test if polyP, as a physiological polymer, might be suitable to treat osteo-articular cartilage and bone defects. Considering the facts that (i) osteoblasts (or osteoblast-related cells) react to polyP with an increased hydroxyapatite mineralization both in vitro and in vivo [10,11], and (ii) chondrocytes with an upregulation of collagen expression in vitro [42,43], we asked if polyP, packed into nano/microparticles, can anabolically influence these cells, osteoblasts, and chondrocytes, in their (close to) natural cell environment, together with their extracellular, three-dimensional matrix. As a model, we used femur explants and incubated those fragments with their bone marrow in vitro. The results revealed under these conditions, which meet the structural, mechanical, chemical, and communicative complexities of the in vivo bone, polyP elicits in MSC and/or osteoblasts an increased expression of *Runx2,* and in MSC and/or chondrocytes an increased expression of *Sox9.* Furthermore, and making this femur explants model even more relevant as an organ-like system, comprising a microenvironment provided with physiological permeability mechanisms for nutrients and growth factors, it is seen that polyP upregulates the expression of the gene encoding the bone-catabolic cathepsin-K, while leaving the expression of the *TRAP* gene uninfluenced. The *TRAP* gene product positively influences both osteoblasts and osteoclasts. This bone anabolic effect displayed by polyP on gene expression level is substantiated by the finding that, also in the femur explants system, the cells respond to polyP with an increased mineralization. Finally, we outlined that polyP can be complexed with ZOL, an approved drug for the prevention and treatment of skeletal complications in patients with bone metastases [23,74], under formation of nano/microparticles, which combine both the cytotoxic effect of ZOL and the mineralization activation potency of polyP. These data might qualify the femur explants system as a model for the screening for and subsequent evaluation of compounds useful for osteo-articular damage repairing therapy. Finally, we outline that the polyP nano/microparticles are versatile baskets for harboring drugs but are also comprising, dependent on the selected counter-ion for polyP used, like Mg^2+^ − Ca^2+^ or Sr^2+^, morphogenetic and (self-)adaptive potencies, which are characteristic of genuine, smart, bio-inspired nano/micro biomaterials.

**Figure 9:**
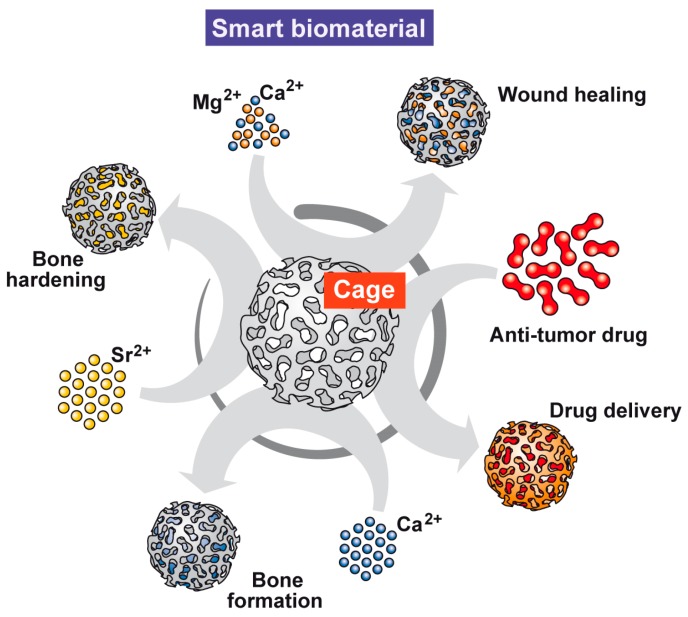
Polyphosphate as a smart genuine nano/micro biomaterial. The introduction of the technology to form amorphous nano-/microparticles under over-stoichiometric ratios between the different cations Mg^2+^ − Ca^2+^ or Sr^2+^ and the polyanion polyP disclosed that Mg^2+^-polyP accelerates wound healing and cartilage formation, Ca^2+^-polyP strongly induces bone formation, and Sr^2+^-polyP, besides inducing bone formation, also hardens the mature hydroxyapatite bone mineral. Furthermore, polyP can act as a basket/cage for encasing drugs, like zoledronic acid.

It has been shown that polyP particles can be incorporated in a straightforward way into pearls or implants, e.g., by embedding them into poly(d,l-lactide-*co*-glycolide) [44,75], which proved firstly, to accelerate the physiological bone regeneration, and secondly, to be superior than β-tricalcium phosphate. In addition, such a scaffold material allows MSC to proliferate. Since, as described in the present study, bone marrow cells in femur explants also show characteristic differentiation towards the osteogenic and chondrogenic lineages, the polyP-based material should turn out to be a useful and promising biomaterial for the repair of osteo-articular defects. With the new data reported here, the polyP-based nano/microparticles have been shown to act as a carrier system for chemotherapeutic agents. In turn, those particles might be qualified for a targeted application in bone tumors and metastases (Figure 9); the polyP polymer acts as a cage for an ionic and hence reversible binding of the respective drug.

Since polyP is a polyanion, it needs a cationic counter-ion for salt formation, paralleled by nano-/microparticle formation. It is highly interesting, and for a (potential) future application in the regenerative implantology very beneficial, that (i) the Mg^2+^ salt of polyP potently stimulates chondrocytes for cartilage formation [43]; also, it strongly promotes wound healing even in diabetic mice [76,77]; (ii) the Ca^2+^ polyP salt accelerates bone formation in vitro as well as in vivo [45]; and (iii), finally, Sr^2+^-polyP elicits an increased healing/mineralization potency on bone defect in vitro as well as in vivo [78]. Accordingly, polyP with its different salt forms can be rated as a genuine and versatile, smart, bio-inspired nano/micro biomaterial (Figure 9).

In conclusion, we are convinced that the physiological polymer polyP can be introduced as a novel biomaterial into the emerging field of custom-designed implants. As outlined, this polymer, either alone or in combination with other anionic mono- or polymers, is suitable for the bio-printing of custom-made, bioactive, tissue-like bone implants. The printing material, bio-ink, can even be supplemented with cells that retain their proliferation capacity, since polyP provides not only the required metabolic fuel but also the phosphate-monomers for the incorporation in the regenerating bone. The number of cells required in those custom-made implants, also fabricated by 3D technology, is comparably low with 5 × 10^5^ cells/mL [46]; usually 6.5 × 10^6^ MSCs/mL are used [79].

## Abbreviations

“Ca-polyP-MP”	Ca-polyP nano/microparticles
“Ca-polyP-ZOL-MP”	calcium polyP-zoledronic acid hybrid nano/microparticles
“Ca-ZOL-MP”	calcium zoledronic acid nano/microparticles
“Na-polyP”	sodium polyP
ALP	alkaline phosphatase
CatK	cathepsin-K
FBS	fetal bovine serum
GAPDH	glyceraldehyde 3-phosphate dehydrogenase
MSC	mesenchymal stem cells
PRP	platelet-rich plasma
qRT-PCR	quantitative real-time polymerase chain reaction
RANKL	receptor activator of the NF-κB ligand
*Runx2*	runt related transcription factor
*Sox9*	sex determining region Y
TRAP	tartrate-resistant acid phosphatase
TRAP^+^	tartrate-resistant acid phosphatase positive
XTT	2,3-bis-(2-methoxy-4-nitro-5-sulfophenyl)-2H-tetrazolium-5-carboxanilide
